# Optimizing outpatient cervical ripening: A mixed‐methods study identifying implementation drivers during a multisite, randomized trial

**DOI:** 10.1002/pmf2.70360

**Published:** 2026-07-14

**Authors:** Rebecca F. Hamm, Anthony C. Sciscione, Brett D. Einerson, Matthew K. Hoffman, Robert M. Silver, Miriam J. Alvarez, Alison G. Cahill, Lisa D. Levine

**Affiliations:** ^1^ Department of Obstetrics & Gynecology Perelman School of Medicine University of Pennsylvania Philadelphia Pennsylvania USA; ^2^ Leonard Davis Institute of Health Economics Philadelphia Pennsylvania USA; ^3^ Department of Obstetrics & Gynecology Christiana Care Health System Newark Delaware USA; ^4^ Department of Obstetrics & Gynecology University of Utah Health Salt Lake City Utah USA; ^5^ Department of Obstetrics & Gynecology Columbia University New York New York New York USA; ^6^ Department of Obstetrics & Gynecology Dell Medical School University of Texas Austin Texas USA

**Keywords:** cervical ripening, Foley catheter, implementation science, labor induction, mixed‐methods, organizational determinants, provider attitudes

## Abstract

**Introduction:**

Data have demonstrated decreased time in the hospital and improved patient satisfaction when using outpatient cervical ripening with a Foley catheter as compared to routine in‐hospital induction. An ongoing multisite trial (the COMFORT trial) is evaluating the impact of outpatient ripening on cesarean delivery, maternal and neonatal morbidity rates. Here, we aimed to characterize provider and organizational level determinants of implementation related to outpatient cervical ripening with a Foley catheter during the COMFORT trial.

**Methods:**

This sequential mixed‐methods study (quan‐ > QUAL; December 2022 to January 2025) was embedded into an ongoing five‐site randomized clinical trial comparing outpatient versus inpatient cervical ripening. Prior to trial initiation, surveys (quan) were distributed to obstetric clinicians at all sites. The survey collected demographics, Foley placement experience, perceived acceptability, and feasibility of outpatient Foley via validated measures [Likert; total 4–20]. One year into the trial, clinician focus groups (QUAL) assessed barriers, facilitators, and sustainability of outpatient Foley, guided by the Consolidated Framework for Implementation Research 2.0 and coded in an integrated approach.

**Results:**

A total of 396 clinicians (64% physicians, 22% nurses, 10% certified nurse midwives [CNMs], 4% nurse practitioners [NPs]/physician assistants [PAs]) completed the pre‐trial survey; 29.2% had previously used outpatient ripening. Acceptability (19/20 interquartile range [IQR] [16–20]) and feasibility (16/20 IQR [15–20]) were high overall but varied by site (*p* = 0.003; *p* = 0.006, respectively). Factors independently associated with acceptability included site, clinician role (physicians/CNMs vs. nurses), and clinician gender. As a result, focus groups were divided by site and role to elicit site‐specific barriers and facilitate open discussion among nurses without physicians/CNMs present. Twelve focus groups (*n* = 33) identified facilitators including belief in the evidence/safety and benefit, as well as leadership support. Barriers included concerns about timely hospital return, perceived patient discomfort, and operational constraints (e.g., space and staffing). In response, participants discussed systematic strategies to promote sustainability, several already enacted, such as standardized patient education and workflow solutions. No meaningful differences in identified barriers, facilitators, or suggested strategies were noted by site or clinician role.

**Conclusion:**

This work identified key determinants influencing the implementation of outpatient cervical ripening with a Foley catheter. Findings (1) allowed for real‐time adaptations to intervention workflow and utilization of new implementation strategies during the course of the multisite trial, and (2) can be utilized to drive success for current and future sites implementing outpatient Foley cervical ripening clinically.

## 1 INTRODUCTION

Induction of labor, which makes up over 30% of all deliveries in the United States, occurs in almost 1.2 million US pregnant patients annually [1, 2]. In addition, with data supporting elective induction at 39 weeks’ gestation [3], induction rates are likely to continue to climb in the coming years [4, 5]. Labor induction, while evidence‐based, contributes to prolonged delivery admission, straining limited inpatient bed capacity, increasing other healthcare costs, and impacting patient experience [3, 6]. Current standard practice in the United States is to begin cervical ripening, the most time‐intensive stage of labor induction, in the inpatient setting [7]. There is a growing interest in implementing labor and delivery interventions that will decrease time in the hospital and improve patient satisfaction, without compromising safety.

Outpatient cervical ripening with a Foley catheter has emerged as a promising intervention to meet this need [8]. Current data, including systematic reviews, support reduced inpatient time and improved patient satisfaction with outpatient Foley for cervical ripening when compared to routine in‐hospital induction [9, 10, 11]. These data have even led to a 2025 practice guideline from the American College of Obstetricians and Gynecologists (ACOG), which states that outpatient cervical ripening is a “safe and effective way to reduce the time from admission to delivery in low‐risk patients” [1]. Yet, gaps in these data remain as prior studies are underpowered to assess the impact on maternal or neonatal outcomes. The COMFORT trial is a large, pragmatic, multicenter randomized trial comparing outpatient Foley ripening to routine inpatient ripening powered to evaluate the impact on cesarean delivery, and safety by measuring maternal and neonatal morbidity, while also evaluating implementation outcomes [clinicaltrials.gov].

Based on the known benefits of outpatient ripening in decreasing time in the hospital and improving patient satisfaction, sites are beginning to implement outpatient Foley into clinical care workflows [12]. These rates may only increase if the COMFORT trial demonstrates improved perinatal outcomes with outpatient Foley. Yet, there is limited evidence to guide outpatient Foley implementation plans and it is likely a driver of the limited uptake of this intervention. Implementation science is the study of optimizing the delivery of evidence‐based practices to the patients who need them [13]. Implementation of any intervention into an obstetric setting is complex as decision‐making in maternal health is generally team‐based and multidisciplinary, including nurses, physicians, and midwives [14]. Organizational context and culture play significant roles in the success of obstetric implementation [15]. As a result, understanding clinician perspectives on maternal health implementation has the potential to catalyze successful incorporation of evidence‐based interventions into practice [16, 17].

The COMFORT trial provides a unique opportunity to examine implementation determinants of outpatient Foley across diverse clinical settings while the intervention is actively being tested for clinical effectiveness. Here, we aimed to assess clinician and organizational‐level barriers and facilitators to outpatient cervical ripening in a mixed‐methods approach, with the overarching goals of (1) allowing for real‐time adaptations to intervention workflow and utilization of new implementation strategies during the course of the multisite trial, and (2) to drive success for current and future sites implementing outpatient Foley cervical ripening clinically.

## 2 METHODS

### 2.1 Parent hybrid type I effectiveness‐implementation randomized clinical trial

This explanatory sequential mixed‐methods (quan → QUAL) study was embedded into a broader type I hybrid effectiveness‐implementation randomized pragmatic clinical trial (the COMFORT trial; clinicaltrials.gov). In this five‐site study, nulliparous women undergoing a term (≥37 weeks) induction of labor wherein the provider intends to use a Foley catheter for cervical ripening are randomized to outpatient cervical ripening with a Foley catheter or routine inpatient cervical ripening. Sites include a mix of birth settings across the United States, including academic and community models, as well as rural and urban environments. The main outcomes of this trial are the cesarean delivery rate as well as the maternal and neonatal morbidity rates. In addition, this trial also planned to assess secondary implementation outcomes relevant to outpatient cervical ripening with a Foley catheter from both the clinician and patient perspectives. This manuscript focuses on the clinician perspective around barriers and facilitators to implementation. We received institutional Review Board approval at each institution for all study procedures; informed consent was obtained prior to initiation of the survey and focus group components of the study. Standards for Reporting Qualitative Research (SRQR) guidelines were utilized in preparing this manuscript [18].

### 2.2 Quantitative methods

Prior to any patient enrollment in the randomized trial, surveys were distributed to obstetric clinicians (physicians, nurses, certified nurse midwives [CNMs], nurse practitioners [NPs], and physician assistants [PAs]) via listserves at all five sites and were open for completion from December 20, 2022 April 12, 2023. In addition to demographic data (age, race, ethnicity, gender, role, specialty, practice characteristics), clinicians were asked to complete (1) questions regarding experience and comfort with placement of Foley and counseling around outpatient Foley, and (2) modified versions of the validated Acceptability of Intervention Measure (AIM), Intervention Appropriateness Measure (IAM), and Feasibility of Intervention Measure (FIM). These three measures are gold‐standard pragmatic approaches to measuring implementation outcomes with strong psychometric properties [19, 20]. All three are four‐item measures scored on a five‐point Likert scale (1 = *strongly disagree*, 5 = *strongly agree*), then either averaged [total score 1–5] or reported as a total [total score 4–20]. For the purposes of this work, all three were modified to our intervention ([Triple P/Implementation Strategy] replaced with “labor induction at home with a Foley catheter (instead of in the hospital)”) (Table S1). Survey results were primarily analyzed using descriptive statistics.

The primary quantitative implementation outcome of this pre‐trial mixed‐methods analysis was the acceptability of outpatient cervical ripening. Thus, a previously described methodology to evaluate implementation acceptability utilizing AIM scores was specifically selected to determine participants for the qualitative portion of the study [21]. As there is no standard cutoff for acceptability utilizing AIM [21], for this analysis, AIM scores <16 out of 20, indicative of at least one response of 3 or less on the Likert scale, encompassed ∼16% of the survey sample and were then deemed “low acceptability” by the investigators. The remainder of the scores were dichotomized as “high acceptability.” In order to determine clinician demographic characteristics that were associated with acceptability, variables were first assessed using bivariate analysis with chi‐square for categorical variables and Wilcoxon rank sum or Student's *t*‐test for continuous variables as appropriate. Clinician race and ethnicity were evaluated due to possible differences in perceptions of intervention acceptability, feasibility, or appropriateness for their practice model or patient population. Those associated at a level *p* < 0.20 were utilized in multivariable logistic regression and considered independently associated if *p* < 0.10 after backwards stepwise elimination. As a result, site, gender, and clinician role were considered as key factors in planning the qualitative focus group portion of the analysis. Parallel analyses were performed for both secondary implementation outcomes, IAM and FIM, but these results were not utilized in planning for the qualitative portion of the study.

### 2.3 Qualitative methods

One year into the overarching randomized trial, clinician focus groups (QUAL) assessed barriers, facilitators, possible mechanisms of effect on clinical outcomes, and sustainability of outpatient Foley. Perceived mechanisms and effect on clinical outcomes were assessed in this study given their likely impact on the acceptability of the intervention itself. Given the quantitative results demonstrating differences in acceptability by site and role (see Section 3), focus groups were divided by site and role to elicit site‐specific barriers and to facilitate open discussion among nurses without physicians and CNMs present. Given the large majority of clinicians identified as female gender, but an association with non‐female gender and lower acceptability of outpatient Foley, purposive sampling by gender was undertaken to ensure the non‐female perspective was obtained. Clinicians were contacted for interest using the same listserves from the quantitative portion of the study. As a result, participants could have been included in both aspects, but completion of the survey was not a requirement for participation in focus groups. We aimed to have at least two focus groups from each of the five sites, one made up of nursing and one of physicians and CNMs. Focus groups were scheduled with 3–5 clinicians. If at the scheduled time at least 2 clinicians were present, the focus group moved forward rather than proceeding with an attempt to reschedule. We determined the sample to be saturated when we ceased to observe novel themes in the answers to each focus group question. Participants in the qualitative portion of the study received a $50 gift card for compensation.

The Consolidated Framework for Implementation Research (CFIR) was used to create the interview guide [22]. A summary of interview questions by CFIR construct is shown in Table 1. Questions were designed to be open‐ended. Focus groups occurred from May 2024 to January 2025 and were conducted over video‐conferencing software and lasted an average of 60 min. One project manager trained in qualitative interviewing led all focus group discussions, regardless of site.

**TABLE 1:** Summary of interview questions, with relationship to Consolidated Framework for Implementation Research (CFIR) constructs.

CFIR domain	CFIR construct	Example interview questions
Innovation	*Evidence strength & quality*	What is your current understanding of the evidence behind outpatient ripening? Were you taught anything about outpatient ripening during your medical training?
	*Relative advantage*	How do you feel outpatient ripening compares to routine inpatient induction? What specifically do you think of how using outpatient Foley in comparison to routine inpatient induction might have an impact on cesarean rate, maternal morbidity, or neonatal morbidity?
	*Adaptability*	How did you overcome any barriers to implementing outpatient ripening? Were adaptations made regarding identifying eligible patients, scheduling, location of placement, availability of trained clinicians, or NST availability?
	*Complexity/Feasibility*	Now that your site is performing outpatient ripening as part of the trial, how easy or difficult do you find the process?
	*General acceptability*	How do you feel generally about outpatient cervical ripening? What do you like about outpatient Foley induction? What don't you like about it?
Outer setting	*Patient needs & resources*	How well do you think outpatient Foley meets the needs of your patients? What do you tell patients about outpatient Foley ripening? Have you heard stories from patients about their experiences at home with the Foley in place?
Inner setting	*Culture*	How do you feel your site's culture supports or doesn't support making changes like outpatient ripening?
	*Readiness for implementation–available resources/leadership engagement*	Who needed to be involved to make outpatient ripening happen at your site? Were there key stakeholders essential to implementation?
Characteristics of individuals	*Knowledge & beliefs about the intervention*	How do you feel about Foley balloons for induction in general? Do you have concerns about patients going home with a Foley in place?
	*Self‐efficacy*	How confident or comfortable do you feel placing or managing outpatient Foley ripening? How confident do you think your colleagues are?
Process	*Engaging*	How were clinicians engaged in implementing outpatient ripening at your site?
	*Reflecting & evaluating*	Do you feel outpatient ripening has impacted patient care once patients return to L&D? Do you think it has impacted patient outcomes? Why or why not?
	*Sustainability/Maintenance*	Outside of the trial, would you continue to use outpatient Foley ripening? Would others at your site? If you moved institutions, would you want it to continue in your practice?

Permission was obtained and all interviews were recorded. Audio from the interviews was transcribed by Datagain Transcription Services (Secaucus, NJ). The transcripts were then uploaded to NVivo 15 software for management and coding. The resulting data were analyzed using an integrated approach encompassing both grounded theory and a priori themes (including CFIR constructs) [23]. Two qualitative research coordinators (C.O. and H.B.) created a codebook, which was then modified based on input from the rest of the study team. The two research coordinators then co‐coded 40% of the transcripts, intentionally checking the codebook against focus groups conducted at different sites, and periodically refined the themes and definitions based on inter‐rater reliability tests in order to facilitate analysis. The two qualitative researchers then each coded half the transcripts independently. The researcher coordinators then synthesized the outputs of the coding and identified the key themes described in this manuscript. Analysis was completed in June 2025.

## 3 RESULTS

### 3.1 Quantitative

There were 396 clinicians, 64% physicians, 22% nurses, 10% CNMs, 4% NPs/PAs, who completed the pre‐trial survey, of which 72.7% (*n* = 288) had previously placed a cervical Foley in any setting (inpatient or outpatient). Of those 288, 256 (88.9%) were comfortable or very comfortable placing a Foley and 29.2% (*n* = 116) had clinical experience with outpatient Foley before the trial. A total of 62.7% (*n* = 249) of the sample felt comfortable or very comfortable counseling around outpatient Foley for cervical ripening. Acceptability, appropriateness, and feasibility were all high with median scores for AIM 19 IQR [16–20], IAM 17 IQR [16–20], and FIM 16 IQR [15–20].

Clinician characteristics compared by “high acceptability” (AIM ≥16/20), which accounted for 84% of clinicians, and “low acceptability” (AIM < 16/20; 16% of clinicians) are shown in Table 2. Clinician characteristics compared by “high perceived appropriateness” (IAM ≥16/20), which accounted for 77% of clinicians, and “low perceived appropriateness” (IAM < 16/20; 23% of clinicians) are shown in Table S1, while comparisons of “high perceived feasibility” (FIM ≥16/20; 73% of clinicians) and “low perceived feasibility” (FIM < 16/20; 27% of clinicians) are shown in Table S2. While site, gender, role, age, practice type, and years of experience were all evaluated in multivariable modeling, only site, gender, and role remained independently associated with acceptability. Site, role, age, and gender were independently associated with appropriateness in multivariable modeling, but only role was independently associated with feasibility. All final models are shown in Table S3.

**TABLE 2:** Demographic characteristics of participants as compared by “high” and “low” acceptability scores related to outpatient ripening with a Foley.

	High acceptability^a^ *n* = 333 *n*(%)	Low acceptability^a^ *n* = 63 *n*(%)	*p* value
Site			0.003
#1	107 (32.1)	29 (46.0)	
#2	58 (17.4)	7 (11.1)	
#3	50 (15.0)	5 (7.9)	
#4	34 (10.2)	14 (22.2)	
#5	84 (25.2)	8 (12.7)	
Role			<0.001
Physician	221 (66.4)	33 (52.4)	
Nurse	66 (19.8)	20 (31.8)	
Certified nurse‐midwife	39 (11.7)	2 (3.2)	
Advanced practice provider	7 (2.1)	8 (12.7)	
Practice type			0.16
Private	19 (7.7)	2 (4.8)	
Faculty‐based outside of hospital	48 (19.4)	10 (23.8)	
Hospital based	167 (67.3)	24 (57.1)	
Other	14 (5.7)	6 (14.3)	0.01
Age (median [IQR])	35 [30–45]	39 [35‐47]	
Gender			<0.001
Female	287 (86.2)	50 (79.4)	
Male	40 (12.0)	5 (7.9)	
Prefer not to say	6 (1.8)	8 (12.7)	
Years of experience			0.063
0–5	142 (42.6)	16 (25.4)	
6–10	56 (16.8)	16 (25.4)	
11–15	49 (14.7)	15 (23.8)	
16–20	31 (9.3)	5 (7.9)	
>20	55 (16.5)	11 (17.5)	
Race			0.35
Black	29 (8.7)	7 (11.1)	
White	266 (79.9)	43 (68.3)	
Asian	20 (6.0)	6 (9.5)	
American Indian	1 (0.3)	0 (0)	
Other/Unknown	17 (5.1)	7 (11.1)	
Ethnicity			0.45
Hispanic or Latinx	19 (5.7)	6 (9.5)	
Not Hispanic or Latinx	311 (93.4)	56 (88.9)	
Prefer not to say	3 (0.9)	1 (1.6)	

^a^ “High acceptability” was defined as Acceptability of Intervention Measure (AIM) score ≥16/20; “low acceptability” was defined as AIM score <16/20.

### 3.2 Qualitative analysis

Twelve focus groups (two to three per site; *n* = 33 total) were held with two to five clinicians per group, including physicians, CNMs, NPs, PAs, trainee physicians (such as residents and fellows), and labor & delivery nurses. Six groups were composed of NPs, PAs, and nurses, while the remaining six encompassed physicians and CNMs. Three of 33 focus group participants (9.0%) identified as male or preferred not to identify their gender. Their experiences ranged from long‐term practitioners with over 20 years in the field to newer clinicians.

### 3.3 Facilitators

Several facilitators of outpatient ripening with a Foley balloon were discussed. First, participants described their comfort with using Foleys, citing standardized training and frequent practice. Several providers also mentioned that their confidence grew with repeated use, even when initial placement presented some challenges. Next, clinicians focused on outpatient Foley's contribution to improving labor and delivery workflows and efficiency. They described the Foley as a simple, low‐resource intervention that allows the shift of the early induction workload out of the hospital, which in turn improves overall unit flow and reduces patient wait times for inpatient beds.

“It's an excellent use of resources for not crowding the L&D unit with people that are just ripening, and it's nice for the patients to not have to be either continually monitored and/or just bothered all night or all day by the routine stuff that has to happen when you're inpatient versus outpatient. And I think for those reasons, I think cost wise and staffing wise, that's one of the huge pluses of it.” (Physician)

Third, clinicians across roles focused on the safety and strength of the evidence behind the procedure, emphasizing their belief that cervical Foley had already been established as a safe intervention.

“They're very, very safe and generally, patients seem to also be onboard and tolerate cervical ripening with a Foley catheter. So, I'm team Foley balloon.” (Certified nurse‐midwife)

In addition, clinicians cited the patient‐centeredness of outpatient Foley as a key facilitator. They referenced that patients receiving outpatient ripening do not have to deal with nurses regularly checking their vitals, and pointed out that patients may feel more control in their home environment.

“From a patient perspective, I think for a lot of patients it's preferable to be at home, it's a lot more comfortable in an environment than a prolonged hospital stay.” (Physician)

A final facilitator to using outpatient cervical ripening was organizational backing. Many providers noted that with the start of the COMFORT trial, there was strong support from leadership and an overall positive culture for starting outpatient Foley balloon procedures.

### 3.4 Barriers

A driving barrier to implementing outpatient Foley ripening was clinicians’ perceptions of the patient experience with pain. Across focus groups, clinicians reported concerns about the pain and discomfort patients would experience, specifically that pain management would be harder outside a hospital.

“The only other concern I would say is pain because some people can tolerate the pain and the cramping, but I can't say the exact numbers of patients that end up requiring either IV medication or end up requiring an epidural before the Foley is out.” (Nurse)

Some clinicians reported they did not want to do outpatient cervical ripening for patients they were worried would not understand when it was time to return to the hospital or did not have access to transportation that could get them to the hospital if their water broke at home. However, clinicians in other health systems felt these issues could be mitigated with enough counseling, anticipatory guidance, and trust in the patients.

“I think that there is a certain amount of patient autonomy that has to be placed into this. Obviously, we send them home with careful instructions. Like, if your water breaks, you need to come back. If you start having vaginal bleeding, you need to come back, things like that. I think some patients do better with following directions than others, but I don't think that's a reason to withhold something like this from them.” (Physician)

Another barrier was concern about bed availability. Several participants stressed that sending patients home with the Foley balloon can free up beds on the labor floor, a clear benefit. At the same time, they worried that if a patient returned and a bed has not been guaranteed, it could lead to poor care. Some providers shared examples of instances where patients had to be placed in emergency or holding areas because no bed was available. Ensuring returning patients are considered and assigned a bed emerged as a critical factor in the potential long‐term success of the intervention.

Finally, clinicians talked about the burden of incorporating new processes into a busy labor unit and overarching resistance to change.

“It's just like one more thing on top of your already crazy schedule. It's like, I'm sorry. I don't actually have time to make room in my brain for [this] study. I'm doing forceps in this other room.” (Physician)

Barriers and facilitators mapped to CFIR constructs are shown in Table 3.

**TABLE 3:** Facilitators and barriers to outpatient ripening with a Foley catheter mapped to Consolidated Framework for Implementation Research (CFIR) constructs.

CFIR domain	CFIR construct	Facilitator/Barrier	Description	Representative quote
Innovation	*Evidence strength & quality*	Facilitator	Belief that Foleys were already established as a safe intervention.	“They're very, very safe and generally.” (CNM)
	*Relative advantage*	Facilitator	Benefits over traditional in‐hospital induction.	“It's an excellent use of resources for not crowding the L&D unit with people that are just ripening… cost wise and staffing wise, that's one of the huge pluses of it.” (Physician)
	*Complexity*	Barrier	Concerns regarding time and mental capacity to add this to the workflow.	“It's just like one more thing on top of your already crazy schedule… I don't actually have time to make room in my brain for [this]study.” (Physician)
Outer Setting	*Patient needs & resources*	Facilitator/Barrier	Facilitator: Beliefs about patient preference. Barrier: Concern that certain patients might not understand when to return or lack transportation if their water broke at home.	Facilitator: “From a patient perspective, I think for a lot of patients it's preferable to be at home. It's a lot more comfortable… than a prolonged hospital stay.” (Physician) Barrier: “Obviously, we send them home with careful instructions. Like, if your water breaks, you need to come back. If you start having vaginal bleeding, you need to come back, things like that. I think some patients do better with following directions than others.” (Physician)
Inner Setting	*Culture/Leadership engagement*	Facilitator	Strong support from leadership and an overall positive culture for outpatient Foley procedures.	“Philosophically, I feel that there's support for ideas like this.” (Nurse)
	*Available resources*	Barrier	Providers worried that if patients returned and a bed was not guaranteed, it could lead to poor care, with some placed in emergency or holding areas.	“They're not active labor. They can just wait, and that's not appropriate. Having them sit in triage for 15 hours is also not appropriate.” (Nurse)
Characteristics of individuals	*Knowledge & beliefs about the intervention*	Facilitator/Barrier	Facilitator: Participants described comfort with Foley placement due to standardized training and frequent practice. Barrier:	Facilitator: “Its not really that different from what we do every day on the labor floor.” (Physician) Barrier: “The only other concern I would say is pain because some people can tolerate the pain and the cramping, but I can't say the exact numbers of patients that end up requiring either IV medication or end up requiring an epidural before the Foley is out.” (Nurse)
	*Self‐efficacy*	Facilitator	Providers noted increased confidence in Foley placement over time, even when initial placement presented challenges.	“I think as this becomes regularly and routinely offered, then it will continue to become easier to work through.” (Physician)

Abbreviation: CNM, certified nurse midwife.

### 3.5 Mechanisms of impact

Focus groups were asked how they thought outpatient Foley was impacting clinical outcomes in their unit, specifically around a possible impact on cesarean delivery rates, and the mechanisms behind those effects. Participants expressed that by transitioning a lengthy part of the induction process to the outpatient setting, it may keep patients from feeling frustrated and “giving up” on the induction process, leading to cesarean birth.

“I think obviously the data will show whether this is true or not, but my perception is, for patients that are coming in and having an outpatient Foley placed, because they have an unripe cervix when they're coming in, that's no longer the case. So my perception is that this is fantastic, because now they're being admitted and we're already that much further into the ripening/induction process. And so I think it can be really disheartening and hard for a patient when they come in and they're getting multiple rounds of misoprostol and a Foley bulb and it's like 16 hours later and they're one centimeter long.” (Physician)

Others discussed that laboring in the comfortable home environment might allow patients more mobility and less stress, thereby contributing to lower cesarean rates.

“I personally think that when people are able to labor at home and have like lower stress hormone levels and be able to be in a comfortable and familiar environment and move around and do what they want to do, that that generally makes things move along faster, or more smoothly.” (Nurse)

Others felt like this intervention was unlikely to impact cesarean rates:

“I could be wrong, but I feel like that's (cesarean rates) pretty much stayed the same. Just because whether or not they get the Foley bulb doesn't usually impact if the baby's going to tolerate labor one way or the other. Also, doesn't impact if they've got a ginormous baby that's just not going to fit out of their pelvis.” (Nurse)

### 3.6 Sustainability

In response to the barriers identified, participants discussed systematic strategies to promote sustainability. First, in response to concerns around perceived patient discomfort and recognition of reasons to return to the hospital, participants recommended thorough pre‐procedure counseling and patient education. By clearly outlining what the outpatient induction process will involve, clinicians believed that patients would be better prepared to cope with associated pressure or even unexpected bleeding. Specific strategies, such as patient‐facing videos or information sheets highlighting patient stories, were suggested. In addition, clinicians proposed standardizing and enhancing clinician counseling with scripting and/or simulation.

“If there's, in the future, some patient testimonials of their experience that might be helpful to share to help patients understand that this is not just safe and appropriate, because their doctor or midwife said so, but that other real patients have gone through the same thing and had a good experience.” (Physician)

In response to concerns around bed management, staffing, and the burden of new processes, there was also discussion about the need for clear protocols developed with the input of all invested parties. Specifics discussed included (1) optimizing the location for placement of the Foley catheter, such as during specific procedure slots in outpatient clinics, in order to minimize staff burden, (2) considering a reserved bed for the patient's inpatient admission, and (3) piloting workflow options to determine what flows best at each location.

Despite the differences in acceptability of this intervention by demographic variables noted in the survey portion of the study, no meaningful differences in identified barriers, facilitators, or suggested strategies were noted by site or clinician role.

## 4 DISCUSSION

In this embedded mixed‐methods study within the COMFORT trial, clinicians across five sites demonstrated high overall acceptability, appropriateness, and feasibility of outpatient cervical ripening with a Foley catheter. Despite broadly favorable perceptions, meaningful heterogeneity in acceptability was observed by site, clinician role, and gender in the quantitative portion of the work. Qualitative findings contextualized survey findings, identifying key barriers and facilitators to the implementation of outpatient Foley. Importantly, clinicians articulated actionable strategies to address these barriers.

Prior work around outpatient Foley has focused on its ability to reduce hospital stays and improve patient satisfaction [9, 10, 11], while the ongoing parent trial discussed here is evaluating its impact on cesarean delivery and maternal and neonatal morbidity rates. Yet, an understanding of the barriers to utilization of this evidence‐based practice and associated derived stakeholder solutions are needed for successful scale. This work expands upon prior single‐site evaluation of the clinician perspective on outpatient ripening [12], to evaluate CFIR‐informed clinician and organizational implementation determinants across a large, multicenter clinician sample. Our work begins to fill the critical gap between evidence and real‐world adoption, particularly as outpatient Foley enters guideline‐supported practice [1].

Notably, participants demonstrated differences in acceptability scores related to outpatient cervical ripening with a Foley by site, gender, and role. Prior studies evaluating perceived implementation climate across inpatient maternity units found both site and role‐based differences [15, 24]. While this work did not specifically uncover why those differences exist, it is reasonable to consider that institutional culture, local leadership engagement, inter‐team dynamics, and varying staffing models could contribute to site, role, or gender based differences in outpatient ripening acceptability.

Facilitators for outpatient ripening focused on clinician familiarity with the intervention and its safety, leadership support for implementation, reduced inpatient burden, and perceived patient‐centered advantages around comfort. Barriers focused on concerns around patient pain and management at home, system constraints, and competing clinical demands. Importantly, barriers were primarily perceptual and logistical, rather than fundamental concerns around safety. The data presented here have already been used to increase the acceptability of the intervention and the surrounding randomized trial, harnessing recommendations from the clinicians actively involved in study procedures. Specifically, a standardized patient education video was created in both English and Spanish. Local multidisciplinary unit leadership was re‐engaged for workflow optimization and bed management planning. At some sites, a patient check‐in process was developed where a clinician calls each patient with a Foley in place at home to assess symptoms, answer questions, and review instructions. These findings not only directly informed study processes but will be critical to any current and post‐trial clinical roll‐outs. Particularly noting site‐based differences, it is reasonable to think that a different suite of implementation strategies may be needed at one site as compared to another, and that any selected implementation strategies may require tailoring for site‐specific needs.

In this study, clinicians hypothesized around mechanisms by which outpatient cervical ripening could potentially improve cesarean delivery rates. These hypothetical pathways, such as through improved patient experience and reduced stress during labor, or more predictable induction trajectories once presenting for admission, are testable and complement the COMFORT trial's effectiveness outcomes. These data represent an example of how clinician insight can inform interpretation of the trial's results.

Strengths of this study include its use of a large, diverse clinician sample across multiple sites. The study uses validated implementation measures in a mixed‐methods design to obtain data with both breadth and depth. Limitations include that this sample is self‐selecting among clinicians at these sites, with survey results that indicated that many already had prior clinical experience with outpatient ripening, and therefore, this may not represent the full range of perspectives. The study was also limited by a small focus group size, with only two to five participants per group. As the decision was made to divide focus groups by site and role to allow for discussions specific to those groups to take place, this approach may have created an echo chamber of role‐specific views or limited interdisciplinary conversations that simulate the real environment of health care provision. Furthermore, the study only focuses on the clinicians’ perspective; however, ongoing mixed‐methods work will explore the patients’ perceptions of outpatient ripening processes as a part of this trial. In addition, implementation context may differ outside academic or trial‐supported settings, limiting generalizability. Future work will evaluate how outpatient ripening is being implemented in the national landscape, aiming to understand what implementation strategies are being utilized across a variety of healthcare contexts.

In conclusion, outpatient cervical ripening with a Foley catheter is viewed by clinicians as acceptable, appropriate, and feasible, but successful implementation depends on addressing identified barriers. By embedding an implementation science–focused evaluation within this effectiveness trial, these findings begin to develop a roadmap for institutions seeking to implement outpatient Foley ripening.

## AUTHOR CONTRIBUTIONS

**Brett D. Einerson**: Supervision; resources; methodology; writing—review and editing; conceptualization; investigation; funding acquisition. **Anthony C. Sciscione**: Conceptualization; investigation; funding acquisition; methodology; writing—review and editing; supervision; resources. **Matthew K. Hoffman**: Conceptualization; investigation; funding acquisition; methodology; writing—review and editing; supervision; resources. **Rebecca F. Hamm**: Conceptualization; investigation; writing—original draft; methodology; writing—review and editing. **Miriam J. Alvarez**: Conceptualization; writing—original draft; writing—review and editing; data curation; supervision; methodology. **Robert M. Silver**: Supervision; resources; methodology; writing—review and editing; conceptualization; investigation; funding acquisition. **Alison G. Cahill**: Conceptualization; investigation; funding acquisition; methodology; writing—review and editing; project administration; supervision; resources. **Lisa D. Levine**: Supervision; resources; project administration; methodology; writing—review and editing; conceptualization; investigation; funding acquisition.

## CONFLICT OF INTEREST STATEMENT

The authors declare no conflicts of interest.

## Supporting information



Supporting Information

Supporting Information

## Data Availability

The data that support the findings of this study are available from the corresponding author upon reasonable request.
