# Supplementary material for: Optimizing outpatient cervical ripening: A mixed‐methods study identifying implementation drivers during a multisite, randomized trial
Source: Pregnancy (Hoboken). 2026 Jul 14;2(4):e70360. doi: 10.1002/pmf2.70360 (PMC13624844; doi:10.1002/pmf2.70360)
Supplement: Supplementary file 1 [file PMF2-2-e70360-s001.docx]

**Supplemental Appendix. Provider Survey Instrument: Perceptions of Outpatient Cervical Ripening**

**Introduction**
Participants were invited to complete a brief survey assessing clinician perceptions of outpatient cervical ripening using a Foley catheter. The survey included demographic, training, experience, and attitudinal items. Responses were self-reported.

**Section 1. Respondent Characteristics**

**1. Do you take care of pregnant patients?**

- Yes
- No

**2. Age**

- Free text response

**3. Race (select all that apply)**

- Black or African American
- White
- Asian
- Latino (not otherwise specified)
- Native Hawaiian or Pacific Islander
- American Indian or Alaska Native
- More than one race
- Other
- Unknown

**4. Ethnicity**

- Hispanic or Latino
- Non-Hispanic or Non-Latino
- Unknown

**5. Gender**

- Cisgender male
- Cisgender female
- Transgender male
- Transgender female
- Non-binary
- Prefer not to answer

**Section 2. Professional Background**

**6. Clinician type**

- Physician
- Nurse
- Midwife
- Nurse Practitioner
- Physician Assistant

**7. Training level**

- Resident
- Fellow
- Attending

**8. Specialty**

- Obstetrics and Gynecology
- Family Medicine
- Maternal-Fetal Medicine
- Other

**9. Location of obstetrical care (select all that apply)**

- Inpatient
- Outpatient

**10. Practice type**

- Private practice
- Faculty-based practice outside the hospital
- Hospital-based practice
- Other (specify)

**11. Primary delivery hospital**

**12. Years in practice (including training)**

- 0–5
- 6–10
- 11–15
- 16–20
- 20

**Section 3. Clinical Experience and Comfort**

**13. Have you ever placed a cervical Foley catheter?**

- Yes
- No

**14. If yes, how comfortable are you placing a cervical Foley catheter?**
(1-5; 1 = Not comfortable, 5 = Very comfortable)

**15. Do you have clinical experience using a Foley catheter for outpatient cervical ripening?**

- Yes
- No

**16. How comfortable are you counseling patients on outpatient cervical ripening?**
(1-5; 1 = Not comfortable, 5 = Very comfortable)

**Section 4. Attitudes Toward Outpatient Cervical Ripening**

Respondents were asked to indicate their level of agreement with the following statements:

**Response scale:**

- Completely disagree
- Disagree
- Neither agree nor disagree
- Agree
- Completely agree

**4A. Acceptability**

- Labor induction at home with a Foley catheter meets my approval.
- Labor induction at home with a Foley catheter is appealing to me.
- I like the idea of labor induction at home with a Foley catheter.
- I welcome labor induction at home with a Foley catheter.

**4B. Appropriateness**

- Labor induction at home with a Foley catheter seems fitting.
- Labor induction at home with a Foley catheter seems suitable.
- Labor induction at home with a Foley catheter seems appropriate.
- Labor induction at home with a Foley catheter applies to my clinical setting.

**4C. Feasibility**

- Labor induction at home with a Foley catheter seems able to be implemented.
- Labor induction at home with a Foley catheter seems possible.
- Labor induction at home with a Foley catheter seems doable.
- Labor induction at home with a Foley catheter seems easy to use.
