# Supplementary material for: Optimizing outpatient cervical ripening: A mixed‐methods study identifying implementation drivers during a multisite, randomized trial
Source: Pregnancy (Hoboken). 2026 Jul 14;2(4):e70360. doi: 10.1002/pmf2.70360 (PMC13624844; doi:10.1002/pmf2.70360)
Supplement: Supplementary file 2 [file PMF2-2-e70360-s002.docx]

Supplementary Table 1: Demographic characteristics of participants as compared by “high” and “low” perceived appropriateness scores related to outpatient ripening with a Foley

|  | | High Perceived Appropriateness ^a^  n=303  n(%) | Low Perceived Appropriateness ^a^  n=91  n(%) | p-value |
| --- | --- | --- | --- | --- |
| **Site** | #1  *#2* | 94 (31.0)  53 (17.5) | 40 (44.0)  11 (12.1) | 0.06 |
|  | *#3* | 44 (14.5) | 12 (13.2) |  |
|  | *#4* | 34 (11.2) | 14 (15.4) |  |
|  | *#5* | 78 (25.7) | 14 (15.4) |  |
| **Role** | *Physician*  *Nurse*  *Certified nurse-midwife*  *Advanced practice provider* | 199 (65.7)  59 (19.5)  39 (12.9)  6 (2.0) | 56 (61.5)  25 (27.5)  2 (2.2)  8 (8.8) | <0.001 |
| **Practice Type** | *Private*  *Faculty-based outside of hospital*  *Hospital based*  *Other* | 17 (7.5)  43 (18.9)  155 (68.0)  13 (5.7) | 4 (6.5)  15 (24.2)  36 (58.1)  7 (11.3) | 0.29 |
| **Age (median [IQR])** |  | 35 [30-45] | 39 [33-47] | 0.058 |
| **Gender** | *Female*  *Male*  *Prefer not to say* | 264 (87.1)  34 (11.2)  5 (1.7) | 70 (76.9)  11 (12.1)  10 (11.0) | <0.001 |
| **Years of Experience** | *0-5*  *6-10*  *11-15*  *16-20*  *>20* | 129 (42.6)  50 (16.5)  48 (15.8)  27 (8.9)  49 (16.2) | 29 (31.9)  21 (23.1)  16 (17.6)  8 (8.8)  17 (18.7) | 0.40 |
| **Race** | *Black*  *White*  *Asian*  *American Indian*  *Other/Unknown* | 28 (9.2)  240 (79.2)  19 (6.3)  1 (0.3)  15 (5.0) | 8 (8.8)  66 (72.5)  7 (7.7)  0 (0.0)  10 (11.0) | 0.19 |
| **Ethnicity** | *Hispanic or Latinx*  *Not Hispanic or Latinx*  *Prefer not to say* | 16 (5.3)  285 (94.1)  2 (0.7) | 9 (9.9)  79 (86.8)  3 (3.3) | 0.04 |
| ^a “^High” perceived appropriateness was defined as Intervention Appropriateness Measure (IAM) score ≥16/20; “Low” perceived appropriateness was defined as IAM score <16/20 | | | | |

Supplementary Table 2: Demographic characteristics of participants as compared by “high” and “low” perceived feasibility scores related to outpatient ripening with a Foley

|  | | High Perceived Feasibility ^a^  n=286  n(%) | Low Perceived Feasibility ^a^  n=108  n(%) | p-value |
| --- | --- | --- | --- | --- |
| **Site** | #1  *#2* | 89 (31.1)  56 (19.6) | 45 (41.7)  8 (7.4) | 0.006 |
|  | *#3* | 41 (14.3) | 15 (13.9) |  |
|  | *#4* | 29 (10.1) | 19 (17.6) |  |
|  | *#5* | 71 (24.8) | 21 (19.4) |  |
| **Role** | *Physician*  *Nurse*  *Certified nurse-midwife*  *Advanced practice provider* | 183 (64.0)  60 (21.0)  38 (13.3)  5 (1.7) | 72 (66.7)  23 (21.3)  3 (2.8)  10 (9.3) | <0.001 |
| **Practice Type** | *Private*  *Faculty-based outside of hospital*  *Hospital based*  *Other* | 17 (8.1)  37 (17.5)  148 (70.1)  9 (4.3) | 4 (5.0)  21 (26.3)  44 (55.0)  11 (13.8) | 0.008 |
| **Age (median [IQR])** |  | 35 [30-45] | 39 [32-46] | 0.10 |
| **Gender** | *Female*  *Male*  *Prefer not to say* | 246 (86.0)  34 (11.9)  6 (2.1) | 88 (81.5)  11 (10.2)  9 (8.3) | 0.02 |
| **Years of Experience** | *0-5*  *6-10*  *11-15*  *16-20*  *>20* | 119 (41.6)  52 (18.2)  43 (15.0)  26 (9.1)  46 (16.1) | 38 (35.2)  20 (18.5)  21 (19.4)  9 (8.3)  20 (18.5) | 0.72 |
| **Race** | *Black*  *White*  *Asian*  *American Indian*  *Other/Unknown* | 22 (7.7)  226 (79.0)  21 (7.3)  1 (0.3)  16 (5.6) | 14 (13.0)  80 (74.1)  5 (4.6)  0 (0.0)  9 (8.3) | 0.20 |
| **Ethnicity** | *Hispanic or Latinx*  *Not Hispanic or Latinx*  *Prefer not to say* | 17 (5.9)  266 (93.0)  3 (1.0) | 8 (7.4)  98 (90.7)  2 (1.9) | 0.65 |
| ^a “^High” perceived feasibility was defined as Feasibility of Intervention Measure (FIM) score ≥16/20; “Low” perceived feasibility was defined as FIM score <16/20 | | | | |

Supplementary Table 3: Multivariable models evaluating associations between clinician demographics and Acceptability of Intervention Measure (AIM), Intervention Appropriateness Measure (IAM), and Feasibility of Intervention Measure (FIM).

| **High Acceptability (Total AIM≥16)** | | | | |
| --- | --- | --- | --- | --- |
| Variable |  | aOR | 95%CI | p-value |
| **Site** | *#1* | REF | -- | -- |
|  | *#2* | 2.20 | 0.86-5.67 | 0.10 |
|  | *#3* | 1.46 | 0.47-4.55 | 0.51 |
|  | *#4* | 0.34 | 0.14-0.84 | 0.02 |
|  | *#5* | 1.80 | 0.65-4.98 | 0.26 |
| **Role** | *Physician* | REF | -- | -- |
|  | *Nurse* | 0.50 | 0.22-1.12 | 0.09 |
|  | *Certified nurse-midwife* | 2.50 | 0.54-11.49 | 0.24 |
|  | *Advanced practice provider* | 0.11 | 0.03-0.39 | 0.001 |
| **Gender** | *Female* | REF | -- | -- |
|  | *Male* | 1.25 | 0.43-3.61 | 0.68 |
|  | *Prefer not to say* | 0.14 | 0.04-0.46 | 0.001 |
| **High Appropriateness (Total IAM≥16)** | | | | |
| **Site** | *#1* | REF | -- | -- |
|  | *#2* | 2.20 | 0.98-4.96 | 0.06 |
|  | *#3* | 1.44 | 0.62-3.36 | 0.40 |
|  | *#4* | 0.75 | 0.33-1.72 | 0.50 |
|  | *#5* | 1.99 | 0.89-4.47 | 0.10 |
| **Role** | *Physician* | REF | -- | -- |
|  | *Nurse* | 0.79 | 0.39-1.58 | 0.50 |
|  | *Certified nurse-midwife* | 5.53 | 1.22-25.01 | 0.03 |
|  | *Advanced practice provider* | 0.35 | 0.10-1.26 | 0.11 |
| **Gender** | *Female* | REF | -- | -- |
|  | *Male* | 1.18 | 0.50-2.78 | 0.70 |
|  | *Prefer not to say* | 0.19 | 0.06-0.63 | 0.006 |
| **Age** |  | 0.97 | 0.95-1.00 | 0.04 |
| **High Feasibility (Total FIM≥16)** | | | | |
| **Role** | *Physician* | REF | -- | -- |
|  | *Nurse* | 1.03 | 0.59-1.78 | 0.93 |
|  | *Certified nurse-midwife* | 4.98 | 1.49-16.66 | 0.009 |
|  | *Advanced practice provider* | 0.25 | 0.08-0.78 | 0.02 |
